# Circulating Skeletal Troponin During Weaning From Mechanical Ventilation and Their Association to Diaphragmatic Function: A Pilot Study

**DOI:** 10.3389/fmed.2021.770408

**Published:** 2021-12-22

**Authors:** Savino Spadaro, Francesca Dalla Corte, Gaetano Scaramuzzo, Salvatore Grasso, Gilda Cinnella, Valentina Rosta, Valentina Chiavieri, Valentina Alvisi, Rosa Di Mussi, Carlo Alberto Volta, Tiziana Bellini, Alessandro Trentini

**Affiliations:** ^1^Department of Translational Medicine, Anesthesia and Intensive Care, University of Ferrara, Ferrara, Italy; ^2^Department of Anesthesia and Intensive Care Medicine, Humanitas Clinical and Research Center-Istituto di Ricovero e Cura a Carattere Scientifico (IRCCS), Milan, Italy; ^3^Department of Emergency and Organ Transplantation, University of Bari, Bari, Italy; ^4^Department of Medical and Surgical Sciences, University of Foggia, Foggia, Italy; ^5^Section of Medical Biochemistry, Molecular Biology and Genetics, Department of Biomedical and Specialist Surgical Sciences, University of Ferrara, Ferrara, Italy

**Keywords:** acute hypoxemic respiratory failure, assisted mechanical ventilation, biomarker, diaphragm, diaphragmatic ultrasound, skeletal troponin, weaning

## Abstract

**Background:** Patients with acute respiratory failure (ARF) may need mechanical ventilation (MV), which can lead to diaphragmatic dysfunction and muscle wasting, thus making difficult the weaning from the ventilator. Currently, there are no biomarkers specific for respiratory muscle and their function can only be assessed trough ultrasound or other invasive methods. Previously, the fast and slow isoform of the skeletal troponin I (fsTnI and ssTnI, respectively) have shown to be specific markers of muscle damage in healthy volunteers. We aimed therefore at describing the trend of skeletal troponin in mixed population of ICU patients undergoing weaning from mechanical ventilation and compared the value of fsTnI and ssTnI with diaphragmatic ultrasound derived parameters.

**Methods:** In this prospective observational study we enrolled consecutive patients recovering from acute hypoxemic respiratory failure (AHRF) within 24 h from the start of weaning. Every day an arterial blood sample was collected to measure fsTnI, ssTnI, and global markers of muscle damage, such as ALT, AST, and CPK. Moreover, thickening fraction (TF) and diaphragmatic displacement (DE) were assessed by diaphragmatic ultrasound. The trend of fsTnI and ssTnI was evaluated during the first 3 days of weaning.

**Results:** We enrolled 62 consecutive patients in the study, with a mean age of 67 ± 13 years and 43 of them (69%) were male. We did not find significant variations in the ssTnI trend (*p* = 0.623), but fsTnI significantly decreased over time by 30% from Day 1 to Day 2 and by 20% from Day 2 to Day 3 (*p* < 0.05). There was a significant interaction effect between baseline ssTnI and DE [*F*_(2)_ = 4.396, *p* = 0.015], with high basal levels of ssTnI being associated to a higher decrease in DE. On the contrary, the high basal levels of fsTnI at day 1 were characterized by significant higher DE at each time point.

**Conclusions:** Skeletal muscle proteins have a distinctive pattern of variation during weaning from mechanical ventilation. At day 1, a high basal value of ssTnI were associated to a higher decrease over time of diaphragmatic function while high values of fsTnI were associated to a higher displacement at each time point.

## Introduction

Ventilatory support is an essential life-saving therapy for intensive care patients with acute respiratory failure (1). However, most patients under mechanical ventilation (MV) experience deleterious impact of mechanical ventilation on the diaphragm (2). The balance between lung and diaphragm protection remains challenging. As known, MV can trigger a sustained change in muscle fibers biochemistry (3), ultimately leading to diaphragmatic atrophy (4). Experimental evidence suggest that signs of increased oxidative stress and diaphragm fiber proteolysis may arise as early as 12 h from MV initiation (5). Ventilator induced diaphragmatic dysfunction (VIDD) (6) has a rapid onset, is related to the duration of ventilation support (7) and affects the clinical outcome (8–11). The diaphragm seems to be more susceptible to fast disuse atrophy, as compared to peripheral skeletal muscles (e.g., pectoralis muscle and latissimus dorsi) (12). In order to minimize VIDD, it has been suggested to implement assisted modes as soon as clinically feasible and safe (13), to minimize patient-ventilator asynchronies (14) and to avoid excessive expiratory braking (15, 16), despite definitive evidence are not available.

In critically ill patients, no bedside tools are available to monitor the muscular function except for ultrasound, which is non-invasive but highly operator-dependent (17). Phrenic nerve stimulation, which would be the “gold standard,” is invasive, complex and thus limited in the routine clinical application.

Specific circulating biomarkers for skeletal muscles have been recently identified. In contrast with non-specific muscular markers, like creatine kinase (CPK), specific markers could potentially open the possibility to provide real time information on the integrity of different types of muscular fibers (18). Slow- and fast-twitch skeletal troponin I (ssTnI and fsTnI) have been shown to be promising markers of damage to slow oxidative (Type I) and fast glycolytic (Type II) muscular fibers (19, 20), respectively.

In a previous report in healthy subjects undergoing an inspiratory threshold loading trial (21), fsTnI was regarded as an early marker, more sensitive than CK, of subclinical diaphragmatic damage. Furthermore, recent data suggest the use of these markers for evaluating subclinical muscular damage (22), since their circulating level are influenced by both muscular mass and amount of muscle disruption.

It is not clear what happens to the circulating levels of skeletal troponin in patients with acute respiratory failure (ARF) undergoing mechanical ventilation, especially during the early phase of weaning. Therefore, the primary aim of the present study was to describe the trend of circulating skeletal troponins during the early part of weaning in a population of mechanically ventilated critically ill patients. Moreover, we hypothesized that values of skeletal troponin beyond normality could be associated to diaphragmatic dysfunction. To test this hypothesis, we compared ultrasound derived diaphragmatic parameters with the baseline levels and the trend of plasma skeletal troponins to determine if they could serve as early markers of diaphragmatic atrophy/ventilator over assistance.

## Methods

### Study Population

This is a longitudinal, single-center, observational cohort study, conducted over a 24-months period (March 2017 to March 2019) in the ICU of the S. Anna University Hospital, Ferrara, Italy. The study was approved by the ethics committee of our institution (Azienda Ospedaliero-Universitaria Ferrara Ethic Committee, number of ethical approval 131084). Informed consent was obtained from each patient or next of kin. All consecutive patients recovering from acute hypoxemic respiratory failure (AHRF) with an expected length of mechanical ventilation of 72 h or more were screened for study inclusion. The inclusion criteria were: age 18 years or older, ventilation in assisted mode, Richmond Agitation Sedation Scale (RASS) between −1 and +1. Exclusion criteria were: history of neuromuscular disease, continuous infusion of muscle-paralyzing agents in the last 48 h, diaphragm atrophy or paralysis, abnormal values of myocardial and muscular damage markers at ICU admission, presence of moderate/severe acute kidney injury (23) at ICU admission, presence of thoracotomy, pneumothorax or pneumo-mediastinum, pregnancy.

### Study Protocol

All patients were enrolled at the beginning of weaning from MV and therefore when able to trigger the ventilator. Specifically, the patients were studied from the 1st (defined Day 1) to the 3rd day (defined Day 3) since the switch from the beginning of assisted mode ventilation (PSV). The following data were collected: mode of mechanical ventilation, ventilator parameters [i.e., pressure support, positive end-expiratory pressure (PEEP), inspired oxygen fraction (FiO_2_)], breathing pattern [i.e., tidal volume (V_T_), respiratory rate (RR)] and the occlusion pressure at 100 ms (P0.1), defined as the negative pressure measured 100 ms after the initiation of an inspiratory effort. All included patients underwent a daily ultrasonographic evaluation of the diaphragmatic function. Arterial blood samples were collected right after ultrasound measurements to evaluate arterial blood gasses and circulating markers. Creatinine was collected each day during the study period to evaluate the onset of moderate/severe acute kidney injury (23).

### Mechanical Ventilation Setting

Patients with ARF were included in the study within 24 h after the initiation of assisted mechanical ventilation (i.e., pressure support ventilation, PSV) according to the attending physicians' judgment. The readiness to sustain PSV was based on the following criteria: (a) improvement of the condition leading to acute respiratory failure, (b) positive end-expiratory pressure (PEEP) lower than 10 cm H_2_O and inspiratory oxygen fraction (FiO_2_) lower than 0.5, (c) Richmond Agitation Sedation Scale (RASS) between −1 and +1, with no sedation or with low dose of continuous infusion of sedation (i.e., propofol 0.5–1.5 mg/kg/h and/or remifentanil 0.03–0.05 mg/kg/min or dexmedetomidine (0.3–1.0 μg/kg/h), (d) ability to trigger the ventilator, (e) hemodynamic stability (with norepinephrine ≤ 0.1 μg/kg/min or equivalent), (f) normothermia.

Pressure support ventilation was set to meet the following targets: V_T_ of 6–8 mL/kg/PBW, with RR 20–30 bpm. Pressure support (PS) was decreased if V_T_ > 8 mL/kg/PBW and/or RR < 20 while it was increases if V_T_ < 6 mL/kg PBW and/or RR > 30 and/or in the presence of respiratory distress (e.g., marked use of the accessory muscles). PEEP and then FiO_2_ were increased if SpO_2_ was < 90%, while FiO_2_ and then PEEP were decreased if SpO_2_ was > 96%. The PEEP and FiO_2_ levels in use before the study were left unchanged. Patients returning into controlled mechanical ventilation due to deteriorating respiratory mechanics or general clinical conditions were excluded from the clinical study.

### Ultrasonography

Ultrasonographic assessments were performed by a single well-trained physician (F.D.C.) by using the same ultrasonography machine (M-Turbo, SonoSite, Inc., USA). All measurements were performed in patients lying in the semi-recumbent position and on the right side. Diaphragmatic excursion (DE) was evaluated using a 3.5 to 5-MHz convex ultrasound probe using a subcostal approach (24–26).

Diaphragmatic thickness and thickening fraction were assessed using a 12-MHz linear ultrasound probe by using an intercostal approach, as previously described (27, 28). Diaphragm thickening fraction (TFdi) was measured in M-mode as TFdi = [(T_EI_-T_EE_)/T_EE_ ] × 100, where T_EE_ and T_EI_ correspond to the thickness of the diaphragm at the end of expiration and inspiration, respectively. Normal diaphragmatic function was defined as the presence of a DE ≥ 10 mm (28) or a TFdi ≥ 30% (29).

### Serum Sampling and Quantification of Skeletal Troponins

Serum samples were obtained from the arterial blood in anticoagulant-free tubes by centrifugation at 1,500 rpm for 10 min after clotting and stored in aliquots at −80°C until assay. To avoid possible loss of bioactivity, samples were analyzed within 3 months from the collection and thawed only once. Slow skeletal Troponin I (ssTnI, Mybiosource, Cat. No. MBS2510383) and fast skeletal Troponin I (fsTnI, Mybiosource, Cat. No. MBS927961) were assayed by commercially available ELISA kits according to manufacturer's instructions. Specific technical details can be found elsewhere (22).

Myoglobin, Creatine Kinase (CPK) and creatinine were determined by routine analysis from the hospital's clinical laboratory. The concentration of aldolase, aspartate aminotransferase (AST), and alanine aminotransferase (ALT) were assayed on undiluted serum samples by coupled spectrophotometric enzymatic assays on a Tecan Infinite M200 (Tecan Group Ltd., Männedorf, Switzerland) as detailed elsewhere (22).

### Demographic and Clinical Data Collection

Demographics, anthropometrics, comorbidities, information, and causes of hospitalization were recorded into study-specific case report forms and database. Simplified Acute Physiology Score II (SAPSII) values, etiology, diagnosis, and severity of AHRF, days on mechanical ventilation before study enrollment were collected for each patient. Sequential organ failure assessment (SOFA) was calculated daily throughout the study observation period. Finally, days of mechanical ventilation, ICU length of stay, hospital length of stay, ICU mortality, and 28-days mortality were recorded as outcome data.

### Statistical Analysis

Given the observational nature of this pilot study we enrolled a convenience sample size of consecutive patients matching inclusion criteria over a 2-year period based on previous studies (30). Continue variables are expressed as mean ± standard deviation or medians [interquartile range] depending on their distribution, whereas categorical variables are presented as frequencies and percentages. The Shapiro-Wilk test was used to assess the assumption of normality. Categorical data were compared using the χ^2^ test or Fisher exact test as appropriate. Unpaired Student's *t*-tests or Mann-Whitney *U*-tests for data with normal or non-normal distribution, respectively, were used to compare continuous variables.

Mixed ANOVA was used to test differences in breathing parameters, diaphragmatic ultrasound measurements and sTnI serum levels among different time points [24 h (Day 1), 48 h (Day 2) and 72 h (Day 3) from assisted mechanical ventilation initiation], after log transformation of variables. In this case, statin use and other variables like sex, age (centered to the mean of 67 years), and BMI (centered to the mean of 28.4 Kg/m^2^) were included in the model as covariates to correct for possible confounding factors. The subjects were divided into two groups based on the presence of higher of lower values than the median of biomarkers at baseline (ssTnI = 66 pg/mL, fsTnI = 31 pg/mL, CK = 68 U/L, myoglobin = 151 ng/mL). Then, we performed a mixed ANOVA by using these groups as between-subject variable to observe the possible interaction between low/high levels of biomarkers at baseline on respiratory effort parameters.

Correlation between diaphragmatic ultrasound measurements and circulating muscle functionality biomarkers were assessed by multivariate linear mixed-effects models, as stated elsewhere (31). sTnI values were tested as predictors of “normal diaphragmatic function” determined by diaphragmatic ultrasound through receiver operator characteristic (ROC) curves. For each ROC curve, sensitivity, specificity, accuracy, and optimal cut-off point using Youden's index were calculated. Statistical analyses were performed using SPSS 20.0 statistical software (SPSS Inc., Chicago, IL). In all statistical analyses, a 2-tailed test was performed and the *p* ≤ 0.05 was considered statistically significant.

## Results

### Patient Population

A total of 62 consecutive patients were included in the study after 1 [1–3] days from ICU admission. The main clinical characteristics of patients at admission are shown in Table 1. Their mean age was 67 ± 13 years old and 43 (69%) were male. Their median SAPSII score was 42 [35–49], resulting in a 29% predicted mortality. The most frequent causes for ICU admission were acute hypoxemic respiratory failure (AHRF) (43%), sepsis (37%), ARDS (24%) and hemorrhagic shock (9%). The median time spent on invasive mechanical ventilation was 10 [6−16] days, and their median ICU length of stay was 13 [8–19] days. No patient developed moderate/severe acute kidney injury during the study period.

**Table 1:** Patients' clinical characteristics at ICU admission.

**Variables**	**Patients (*N* = 62)**
Age, years	67 ± 13
Male sex, *n* (%)	43 (69)
BMI, kg/m^2^	28.4 ± 5.5
SAPS II score at admission	42 [35–49]
Smoker, *n* (%)	
Actual	18 (29)
Former	6 (10)
Comorbidities, *n* (%)	
Heart diseases	16 (26)
Hypertension	37 (60)
Chronic cardiac ischemia	7 (11)
COPD	9 (15)
Diabetes	13 (21)
CKD	5 (8)
Reason for MV initiation, *n* (%)	
AHRF	29 (47)
Sepsis	6 (10)
Septic shock	18 (29)
Hemorrhagic shock	4 (6)
Coma	4 (6)
Cardiogenic shock	1 (2)
Outcomes	
Days spent on MV	10 [6–16]
ICU LOS	13 [8–19]
Hospital LOS	31 [15–53]
28-days mortality	10 (16)

*BMI, body mass index, SAPS, simplified acute physiology score, COPD, chronic pulmonary obstructive disease, CKD, chronic kidney disease, AHRF, acute hypoxemic respiratory failure, ICU, intensive care unit, LOS, length of stay*.

### Skeletal Troponin and “Classic” Muscle Damage Parameters

**Figure 1:**
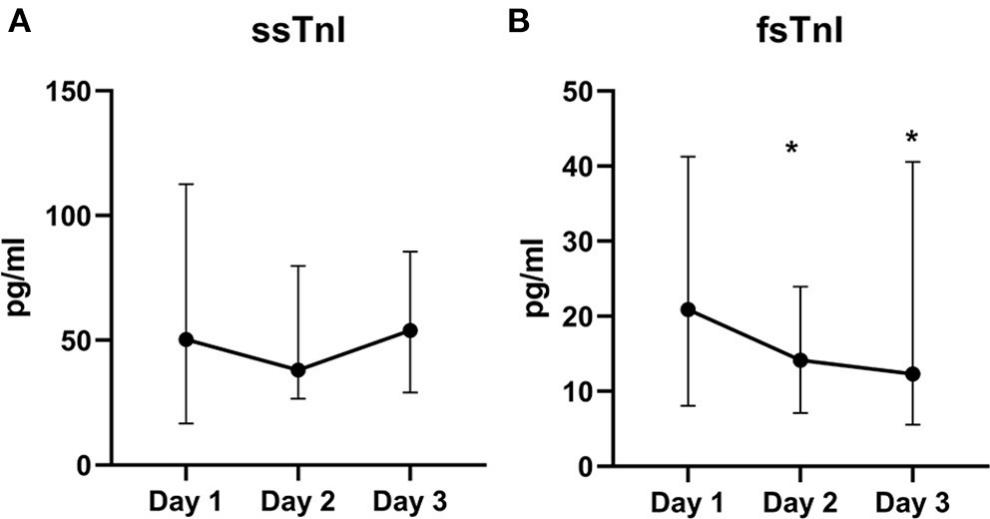
Skeletal TnI serum levels over the study period. Value of ssTnI **(A)** were not different over time, whereas fsTnI **(B)** significantly decreased within the time frame of the study (*p* < 0.05 for within-subjects linear trend). The dots represent the median whereas the upper and lower bars the upper and lower 95% confidence interval, respectively. *p < 0.05 vs. T0.

We did not find significant variations in the ssTnI trend (*p* = 0.623). On the contrary, fsTnI significantly decreased over time by 30% from Day 1 to Day 2 and by 20% from Day 2 to Day 3, *p* < 0.05, within-subjects linear contrast: *p* < 0.05 (Table 2 and Figure 1). Of note, by correcting the values for the use of statin as a confounding factor, fsTnI still significantly decreased over time (*p* < 0.05). However, when considering the other confounding factors (sex, centered age, and centered BMI), the fsTnI trend was not significant.

**Table 2:** Skeletal troponin and “traditional” muscle damage parameters during the study period.

**Variables**	**Day 1**	**Day 2**	**Day 3**	***p*-value**
ssTnI, pg/mL	66 [15–164]	55 [18–140]	51 [18–154]	0.623
fsTnI, pg/mL	31 [5–90]	18 [5–76]	13 [2–67]	<0.05
CK, U/L	68 [26–243]	55 [21–176]	44 [15–104]	<0.0001
Myoglobin, ng/mL	151 [57–276]	85 [40–153]	79 [41–126]	<0.0001
AST, U/L	5.4 [1.6–10.9]	3.4 [1.3–9.7]	3.9 [1.3–10.6]	0.226
ALT, U/L	4.8 [2.1–20.8]	5.3 [2.2–13.8]	6.1 [2.2–13.2]	0.617
Aldolase, U/L	4.5 [3.2–5.7]	3.9 [3.3–5.6]	4.2 [3.4–5.7]	0.789

*ssTnI, slow skeletal troponin I, fsTnI, fast skeletal troponin I, AST, aspartate aminotransferase, ALT, alanine aminotransferase, CK, creatine kinase*.

A decreasing trend over time was also detected for myoglobin (*p* < 0.0001), with a 45% decrease from Day 1 to Day 2 and almost a 30% decrease from Day 2 to Day 3. Similar data were also observed for CPK, which showed a significant decrease over time (Table 2, *p* < 0.0001) with almost 20% lower values each following day. None of the other traditional markers (aldolase, AST, and ALT) showed significant variations within the study. Of note, by correcting the values of both myoglobin and CPK for statin use the variables remained significant (*p* = 0.011 and *p* < 0.001, respectively), whereas they lose their significance in the fully corrected model.

### Ventilation Parameters and Clinical Variables Over Time

Respiratory parameters and gas exchange remained stable throughout the study. The number of patients requiring the use of vasoactive drugs decreased over time (Day 1: 48%, Day 2: 37%, Day 3: 29%, Table 3).

**Table 3:** Clinical and mechanical ventilation parameters during the study period.

	**Day 1**	**Day 2**	**Day 3**	***p*-value**
Pressure support, cmH_2_O	9 ± 4	9 ± 3	8 ± 4	0.036
PEEP applied, cmH_2_O	8 ± 3	8 ± 3	8 ± 3	0.211
Respiratory rate, bpm	15 ± 5	16 ± 6	17 ± 5	0.548
V_E_, L/min	7.5 ± 2.0	7.5 ± 2.0	8.1 ± 2.1	0.418
Cdyn, ml/cmH_2_O	59 [40–70]	53 [39–64]	56 [40–74]	0.433
PaO_2_/FiO_2_	228 [156–293]	208 [153 −305]	230 [153 −280]	0.799
MAP, mmHg	83 [75–95]	85 [73–96]	83 [76–93]	0.740
SOFA score	6 [3–8]	5 [3–7]	4 [3–7]	0.852
Vasoactive drug use, *n* (%)	30 (48)	23 (37)	18 (29)	-
Steroid use, *n* (%)	26 (42)	29 (47)	26 (42)	-

*PEEP, positive end-expiratory pressure, V_E_, minute ventilation, Cdyn, dynamic compliance, PaO_2_/FiO_2_, oxygen partial arterial tension/inspired oxygen fraction, MAP, mean arterial pressure, SOFA, sequential organ failure assessment*.

### Skeletal Troponin and Diaphragmatic Ultrasound

**Figure 2:**
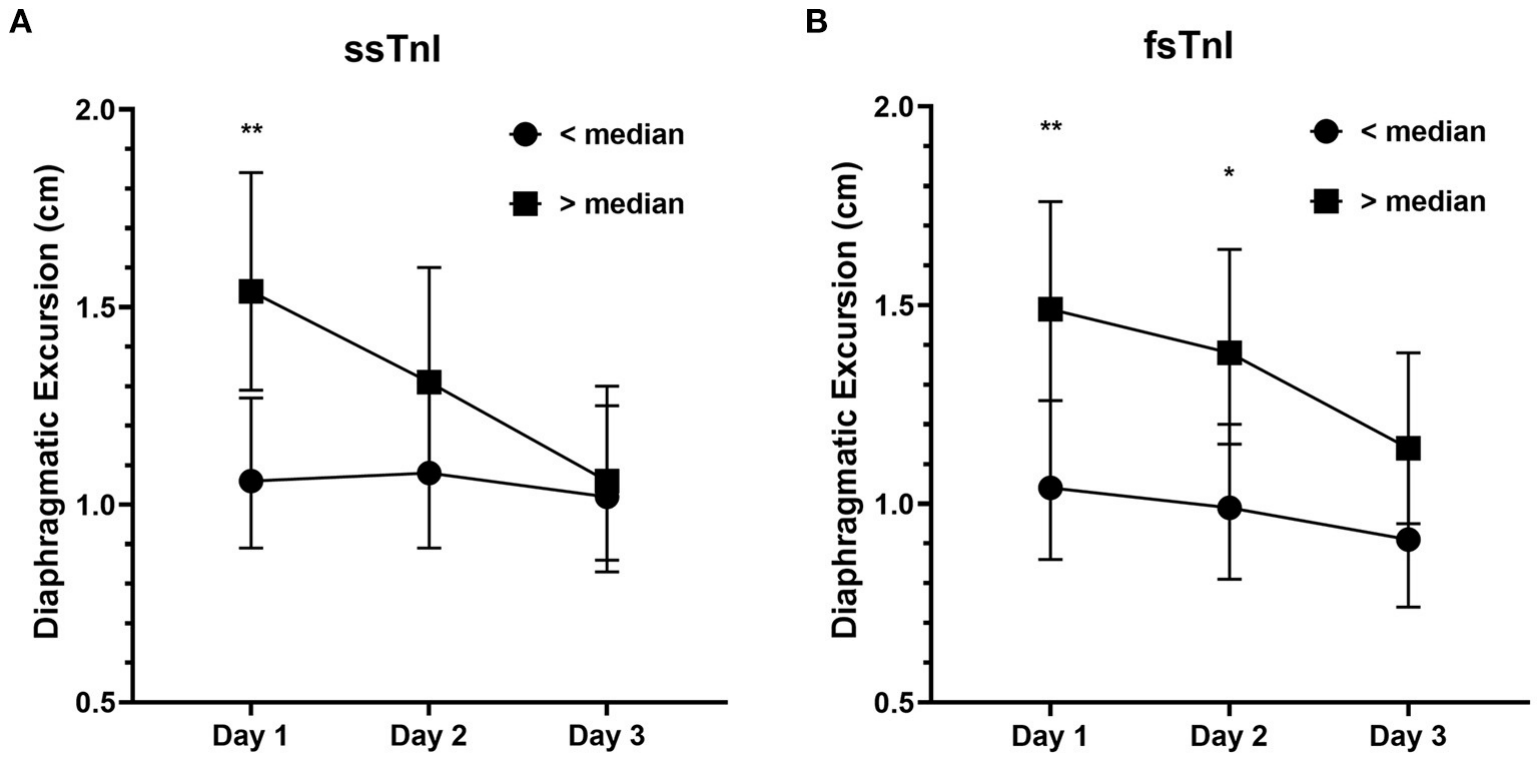
Diaphragmatic excursion (cm) in patients with high or low median levels of skeletal troponins. **(A)** The decrease in diaphragmatic excursion was more prominent in patients with higher basal levels of ssTnI (i.e., >median) denoting the influence of ssTnI basal levels on DE. On the contrary, fsTnI basal levels **(B)** did not influence the decrease in DE. The dots represent the geometric means whereas the upper and lower bars the upper and lower 95% confidence interval, respectively. **p < 0.01, *p < 0.05.

We found a significant interaction effect between baseline ssTnI levels and DE [*F*_(2)_ = 4.396, *p* = 0.015], indicating that the observed decrease in DE over time was dependent on the baseline levels of ssTnI. In particular, subjects with high basal levels of ssTnI had a greater decrease in DE when compared to those with low ssTnI basal levels, identified by the steeper slope in Figure 2A. We did not find any significant interaction between baseline fsTnI levels and the DE trend [*F*_(2)_ = 0.662, *p* = 0.518, Figure 2B]. However, contrary to what observed for ssTnI, patients characterized by high basal levels of fsTnI had a higher DE at each time point than those with low fsTnI basal levels (log transformed variables: Day 1 mean difference: −0.356, *p* = 0.006, Day 2: −0.332, *p* = 0.014, Day 3: −0.102, *p* = 0.102). CK and myoglobin, did not any correlation with DE despite their decrease over time (data not shown). The TFdi trend was not influenced by the baseline levels of any of the measured muscular biomarkers.

Finally, we evaluated whether the frequency of patients developing diaphragmatic dysfunction (defined as DE < 1 cm at Day 3) was different between those demonstrating higher or lower levels of muscular biomarkers at Day 1 (i.e., higher or lower of the median serum concentration). The frequency of patients developing diaphragmatic dysfunction at Day 3 was independent from both ssTnI (40.6% of subjects each group, Fisher's exact test, *p* = 1), fsTnI (low fsTnI vs. high fsTnI: 47.1 vs. 35.3%, *p* = 0.460) (Supplementary Figure 1), CPK or myoglobin. When diaphragmatic dysfunction was evaluated by TFdi (i.e., <30%), the results were similar. We did not find any significant correlation between inspiratory effort parameters and muscular biomarkers within-subjects (e.g., at each time point, Supplementary Table 1). The only exception was for myoglobin, which showed a positive within-subjects relationship with both DE and TFdi (*r* = 0.464, *p* = 0.001 and *r* = 0.462, *p* = 0.001, respectively). Interestingly, ssTnI and fsTnI showed a significant between-subjects positive correlation with DE and TEE (for ssTnI *r* = 0.332, *p* = 0.038 for DE and *r* = 0.346, *p* = 0.027 for TEE and for fsTnI *r* = 0.445, *p* = 0.005 for DE and *r* = 0.400, *p* = 0.009 for TEE). As such, this indicates that subjects with higher serum levels of skeletal troponins had higher DE and TEE values. Instead, CK correlated only with TEE (*r* = 0.381, *p* = 0.016) and P0.1 (*r* = 0.657, *p* = 0.002).

### Skeletal Troponins as Diaphragmatic Function Predictors

**Figure 3:**
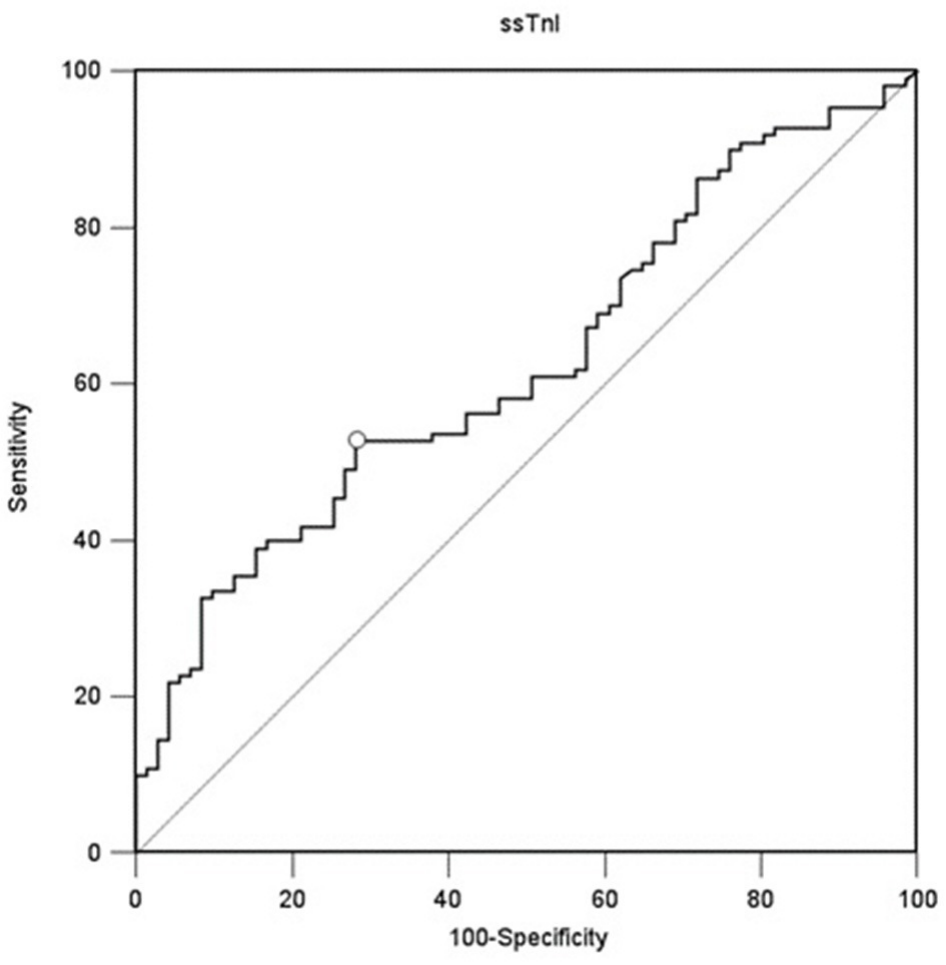
ROC curve analysis for ssTnI as a predictor of normal diaphragmatic function as assessed by diaphragmatic ultrasound (diaphragmatic displacement ≥ 10 mm).

**Figure 4:**
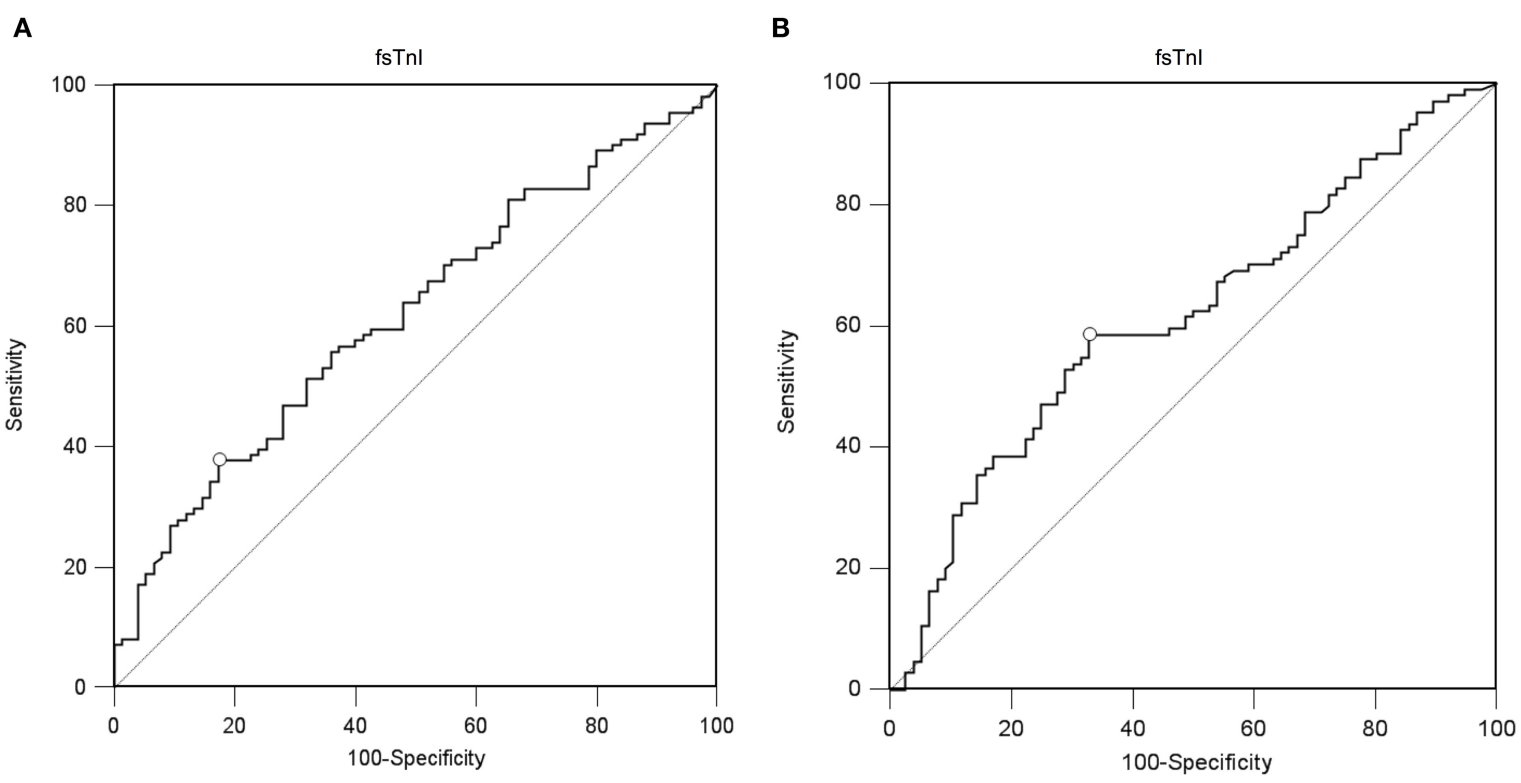
ROC curve analysis for fsTnI as a predictor of normal diaphragmatic function as assessed by diaphragmatic ultrasound (**A**, diaphragmatic excursion ≥ 10 mm, **B**, diaphragmatic thickening fraction ≥ 30%).

A value of ssTnI > 60.06 pg/mL was found to detect a DE ≥ 10 mm (AUC-ROC = 0.628, 95% CI = 0.551 to 0.696, *p* = 0.003) with sensibility 53 [43–62] % and specificity 72 [60–82] % (Figure 3). A value of fsTnI > 46.81 pg/mL was found to detect a DE ≥ 10 mm (AUC-ROC = 0.619, 95% CI = 0.545 to 0.689, *p* = 0.004) with sensibility 38 [29–48] % and specificity 83 [72–90] % (Figure 4A). A value of fsTnI > 18.29 pg/mL was found to detect a TFdi ≥ 30% (AUC-ROC = 0.617, 95% CI = 0.541 to 0.688, *p* = 0.006) with sensibility 59 [49–68] % and specificity 67 [55–76] % (Figure 4B).

## Discussion

In this study we described the trend of circulating skeletal troponin in a population of mechanical ventilated ICU patients during the early phase of weaning from mechanical ventilation. We found that (1) the fast but not the slow isoform of skeletal troponin decreased over time within the first 3 days of weaning, (2) patients with higher levels of ssTnI on day 1 had a higher decrease of diaphragmatic excursion while (3) patients with higher basal fsTnI had higher DE (4) both fsTnI and ssTnI showed a significant positive between-patients correlation with both DE and TFdi while no correlation was found between myoglobin and CPK levels and ultrasound-derived parameters.

Critical illness-associated diaphragm weakness can affect up to 64% of patients within 24 h after intubation (32) and up to 80% of patients requiring prolonged mechanical ventilation (33). However, to the best of our knowledge, no serum biomarker has been suggested to specifically diagnose and monitor the development of respiratory muscles damage. A major limitation of traditional serum biomarkers (i.e., CK, lactate dehydrogenase, myoglobin, AST) is that they have a large normal reference range for healthy subjects (19) and thus, low levels of injury in the individual patient may go undetected. In addition, although these markers are useful for the study of muscle-related diseases, they suffer from a low specificity in detecting solely the damage to skeletal muscles, since an increase of these proteins may also be related to myocardial injury (34).

We hereby describe for the first time the trend of circulating skeletal troponin during the early phase of weaning. We found that the fast skeletal troponin decreased over time during the 3 days of observation but not the slow isoform. The discrepancy between the circulating values of these two isoforms is not new and has been recently found in patients with neuromuscular disease (35). Elevations in both ssTnI and fsTnI have been recorded in severe trauma and ischemia (36), therefore suggesting that the increase of ssTnI into circulation may require muscle injury beyond the damage caused by defective contraction or, in our case, by mechanical ventilation. We might then speculate that an increase in fsTnI could be a marker of muscle overload, rather than a sign of loss of muscular mass caused by ischemia or direct muscular trauma. At the same time, the very decrease of ssTnI below the “normal threshold” could indicate a diaphragm dysfunctionality or muscular mass loss, given the found relationship between DE and ssTnI.

In fact, both fsTnI and ssTnI, but not the other classical markers of muscular damage, were positively correlated with ultrasonographic measurements of diaphragmatic displacement. In particular, subjects with higher levels of these two markers had both higher diaphragmatic excursion and diaphragmatic thickness at end-expiration.

Recently, Dres et al. (37) showed that the use of parasternal intercostal muscle ultrasound was responsive to respiratory load and a greater parasternal intercostal muscle thickening under pressure support ventilation was associated with diaphragm dysfunction. Accordingly, it could be interesting to investigate the relative contribution of parasternal intercostal muscle in relationship of skeletal troponin kinetics.

Our study population seemed to maintain clinical stability over time, with stable levels of mechanical ventilation assistance (i.e., pressure support and PEEP) and no substantial changes in respiratory mechanics and gas exchange. The values of P0.1 (ranging between 1.4 and 1.2) were relatively low and both median values of diaphragmatic excursion and thickening fraction were below the thresholds for diaphragmatic weakness (9, 28, 38) indicating that a “diaphragm protective” mechanical ventilation was maintained (39). Nevertheless, we found an increasing trend of patients presenting a diaphragmatic dysfunction, as assessed by ultrasound, over time. These findings prompt the need of a continuous and accurate monitoring of respiratory muscle function during the ICU stay (40).

Diaphragmatic ultrasound is a non-invasive tool to assess the function of the diaphragm, but the technique is time consuming and highly operator dependent. Therefore, dosing fsTnI and ssTnI could potentially increase the number of patients screened for diaphragmatic dysfunction, improve the use of human resources, and allow this assessment also when and where an expert sonographer is not available.

Our study has relevant limitations. First, it is a single-center designed study with a small sample size. Nevertheless, this is a pilot study aiming at describing for the first time the trend of a novel serum marker in a general ICU population of patients recovering from AHRF and our study population might be used as a reference for normal values for a general ICU population during the early phases of their ICU stay. Second, we compared serum skeletal troponin levels only to diaphragmatic dysfunction, without taking into account global muscular mass or performing direct measurements of limb function. However, since the diaphragm is more sensitive to iatrogenic injury caused by MV (12), we decided to focus on this peculiar muscle after a relatively short period of controlled mechanical ventilation (i.e., 1 [1–3] days). Further studies are needed to assess whether the two isoforms of skeletal troponin are associated to systemic muscle wasting, sarcopenia and extradiaphragmatic wasting during ICU stay (41). Third, patients' population (i.e., patients recovering from AHRF from several causes) is representative of a general ICU population, but little is known on patients' diaphragmatic function before ICU admission. Fourth, we have followed patients for a relatively short time compared to their median ICU length of stay (i.e., 13 [8–19] days), the latter issue is related to the need to perform a time consuming, expensive and not readily available analysis for skeletal troponin serum levels. Finally, we have not studied patients both during controlled mechanical ventilation and during unassisted spontaneous breathing. Further studies are needed to assess skeletal troponin trend during the whole respiratory failure treatment, from complete assistance to complete weaning from mechanical ventilation.

## Conclusions

Circulating fast and slow skeletal troponin have specific and different trends in the early phase of weaning from mechanical ventilation and they correlate with ultrasound derived diaphragmatic assessment parameters. The fsTnI decreased during the early phase of weaning while high initial values of ssTnI are associated to a higher decrease of diaphragmatic displacement over time. Further studies are needed to confirm the relationship between these novel biomarkers, protective mechanical ventilation and weaning outcome.

## Data Availability Statement

The raw data supporting the conclusions of this article will be made available by the authors, without undue reservation.

## Ethics Statement

The studies involving human participants were reviewed and approved by Ethics Committee of Ferrara. The patients/participants provided their written informed consent to participate in this study.

## Author Contributions

SS, AT, FD, SG, TB, and CV were involved in the conception and the design of the study, analyzed the data, and wrote the paper. VC, VR, and VA collected the data. FD performed the statistical work. RD, GC, AT, and FD contributed to the analysis of the data, SS, FD, AT, SG, GC, TB, and CV contributed to the critical revision of the manuscript for important intellectual content. All authors read and approved the final manuscript.

## Funding

This work was supported by the grant number GR-2013-023555391: Diaphragmatic dysfunction in critically ill patients undergoing mechanical ventilation from the Italian Ministry of Health (Bando Ricerca Finalizzata 2013).

## Conflict of Interest

The authors declare that the research was conducted in the absence of any commercial or financial relationships that could be construed as a potential conflict of interest.

## Publisher's Note

All claims expressed in this article are solely those of the authors and do not necessarily represent those of their affiliated organizations, or those of the publisher, the editors and the reviewers. Any product that may be evaluated in this article, or claim that may be made by its manufacturer, is not guaranteed or endorsed by the publisher.

## References

[1] Brochard L Slutsky A Pesenti A. Mechanical ventilation to minimize progression of lung injury in acute respiratory failure. Am J Respir Crit Care Med. (2017) 195:438–42. 10.1164/rccm.201605-1081CP 27626833

[2] Di Mussi R Spadaro S Mirabella L Volta CA Serio G Staffieri F. Impact of prolonged assisted ventilation on diaphragmatic efficiency: NAVA versus PSV. Crit Care. (2016) 20:1. 10.1186/s13054-015-1178-0 26728475 PMC4700777

[3] Jaber S Petrof BJ Jung B Chanques G Berthet J-P Rabuel C. Rapidly progressive diaphragmatic weakness and injury during mechanical ventilation in humans. Am J Respir Crit Care Med. (2011) 183:364–71. 10.1164/rccm.201004-0670OC 20813887

[4] Levine S Nguyen T Taylor N Friscia ME Budak MT Rothenberg P. Rapid disuse atrophy of diaphragm fibers in mechanically ventilated humans. N Engl J Med. (2008) 358:1327–35. 10.1056/NEJMoa070447 18367735

[5] Hudson MB Smuder AJ Nelson WB Bruells CS Levine S Powers SK. Both high level pressure support ventilation and controlled mechanical ventilation induce diaphragm dysfunction and atrophy. Crit Care Med. (2012) 40:1254–60. 10.1097/CCM.0b013e31823c8cc9 22425820 PMC3308123

[6] Vassilakopoulos T Petrof BJ. Ventilator-induced diaphragmatic dysfunction. Am J Respir Crit Care Med. (2004) 169:336–41. 10.1164/rccm.200304-489CP 14739134

[7] Hermans G Agten A Testelmans D Decramer M Gayan-Ramirez G. Increased duration of mechanical ventilation is associated with decreased diaphragmatic force: a prospective observational study. Crit Care. (2010) 14:R127. 10.1186/cc9094 20594319 PMC2945090

[8] Goligher EC Dres M Patel BK Sahetya SK Beitler JR Telias I. Lung- and diaphragm-protective ventilation. Am J Respir Crit Care Med. (2020) 202:950–61. 10.1164/rccm.202003-0655CP 32516052 PMC7710325

[9] Goligher EC Dres M Fan E Rubenfeld GD Scales DC Herridge MS. Mechanical ventilation-induced diaphragm atrophy strongly impacts clinical outcomes. Am J Respir Crit Care Med. (2018) 197:204–13. 10.1164/rccm.201703-0536OC 28930478

[10] Spadaro S Grasso S Mauri T Dalla Corte F Alvisi V Ragazzi R. Can diaphragmatic ultrasonography performed during the T-tube trial predict weaning failure? The role of diaphragmatic rapid shallow breathing index. Crit Care. (2016) 20:305. 10.1186/s13054-016-1479-y 27677861 PMC5039882

[11] Di Mussi R Spadaro S Volta CA Bartolomeo N Trerotoli P Staffieri F. Continuous assessment of neuro-ventilatory drive during 12 h of pressure support ventilation in critically ill patients. Crit Care. (2020) 24:652. 10.1186/s13054-020-03357-9 33218354 PMC7677450

[12] van Hees HWH Schellekens WJM Andrade Acuña GL Linkels M Hafmans T. Titin and diaphragm dysfunction in mechanically ventilated rats. Intensive Care Med. (2012) 38:702–9. 10.1007/s00134-012-2504-5 22327561 PMC3308006

[13] Jubran A Grant BJB Laghi F Parthasarathy S Tobin MJ. Weaning prediction: esophageal pressure monitoring complements readiness testing. Am J Respir Crit Care Med. (2005) 171:1252–9. 10.1164/rccm.200503-356OC 15764727

[14] Mojoli F Iotti GA Arnal J-M Braschi A. Is the ventilator switching from inspiration to expiration at the right time? Look at waveforms! *Intensive Care Med*. (2016) 42:914–5. 10.1007/s00134-015-4174-6 26690075

[15] Pellegrini M Hedenstierna G Roneus A Segelsjö M Larsson A Perchiazzi G. The diaphragm acts as a brake during expiration to prevent lung collapse. Am J Respir Crit Care Med. (2017) 195:1608–16. 10.1164/rccm.201605-0992OC 27922742

[16] Volta CA Dalla Corte F Ragazzi R Marangoni E Fogagnolo A Scaramuzzo G. Expiratory flow limitation in intensive care: prevalence and risk factors. Crit Care. (2019) 23:395. 10.1186/s13054-019-2682-4 31806045 PMC6896682

[17] Dres M Goligher EC Dubé B-P Morawiec E Dangers L Reuter D. Diaphragm function and weaning from mechanical ventilation: an ultrasound and phrenic nerve stimulation clinical study. Ann Intensive Care. (2018) 8:53. 10.1186/s13613-018-0401-y 29687276 PMC5913054

[18] Simpson JA Labugger R Hesketh GG D'Arsigny C O'Donnell D Matsumoto N. Differential detection of skeletal troponin I isoforms in serum of a patient with rhabdomyolysis: markers of muscle injury? Clin Chem. (2002) 48:1112–4. 10.1093/clinchem/48.7.1112 12089186

[19] Sorichter S Mair J Koller A Gebert W Rama D Calzolari C. Skeletal troponin I as a marker of exercise-induced muscle damage. J Appl Physiol. (1997) 83:1076–82. 10.1152/jappl.1997.83.4.1076 9338413

[20] Chapman DW Simpson JA Iscoe S Robins T Nosaka K. Changes in serum fast and slow skeletal troponin I concentration following maximal eccentric contractions. J Sci Med Sport. (2013) 16:82–5. 10.1016/j.jsams.2012.05.006 22795680

[21] Foster GE Nakano J Sheel AW Simpson JA Road JD Reid WD. Serum skeletal troponin I following inspiratory threshold loading in healthy young and middle-aged men. Eur J Appl Physiol. (2012) 112:3547–58. 10.1007/s00421-012-2337-5 22323298

[22] Trentini A Spadaro S Rosta V Manfrinato MC Cervellati C Dalla Corte F. Fast skeletal troponin I, but not the slow isoform, is increased in patients under statin therapy: a pilot study. Biochem Med. (2019) 29:010703. 10.11613/BM.2019.010703 30591813 PMC6294157

[23] Kellum JA Lameire N KDIGO AKI Guideline Work Group. Diagnosis, evaluation, and management of acute kidney injury: a KDIGO summary (Part 1). Crit Care. (2013) 17:204. 10.1186/cc11454 23394211 PMC4057151

[24] Spadaro S Grasso S Dres M Fogagnolo A Dalla Corte F Tamburini N. Point of care ultrasound to identify diaphragmatic dysfunction after thoracic surgery. Anesthesiology. (2019) 131:266–78. 10.1097/ALN.0000000000002774 31166236

[25] Goligher EC Laghi F Detsky ME Farias P Murray A Brace D. Measuring diaphragm thickness with ultrasound in mechanically ventilated patients: feasibility, reproducibility and validity. Intensive Care Med. (2015) 41:642–9. 10.1007/s00134-015-3687-3 25693448

[26] Umbrello M Formenti P Longhi D Galimberti A Piva I Pezzi A. Diaphragm ultrasound as indicator of respiratory effort in critically ill patients undergoing assisted mechanical ventilation: a pilot clinical study. Crit Care. (2015) 19:161. 10.1186/s13054-015-0894-9 25886857 PMC4403842

[27] Vivier E Mekontso Dessap A Dimassi S Vargas F Lyazidi A Thille AW. Diaphragm ultrasonography to estimate the work of breathing during non-invasive ventilation. Intensive Care Med. (2012) 38:796–803. 10.1007/s00134-012-2547-7 22476448

[28] Kim WY Suh HJ Hong S-B Koh Y Lim CM. Diaphragm dysfunction assessed by ultrasonography: influence on weaning from mechanical ventilation. Crit Care Med. (2011) 39:2627–30. 10.1097/CCM.0b013e3182266408 21705883

[29] DiNino E Gartman EJ Sethi JM McCool FD. Diaphragm ultrasound as a predictor of successful extubation from mechanical ventilation. Thorax. (2014) 69:423–7. 10.1136/thoraxjnl-2013-204111 24365607

[30] Schepens T Verbrugghe W Dams K Corthouts B Parizel PM Jorens PG. The course of diaphragm atrophy in ventilated patients assessed with ultrasound: a longitudinal cohort study. Crit Care. (2015) 19:422. 10.1186/s13054-015-1141-0 26639081 PMC4671211

[31] Hoffman L. Longitudinal Analysis: Modeling Within-Person Fluctuation and Change. Routledge (2015).

[32] Demoule A Jung B Prodanovic H Molinari N Chanques G Coirault C. Diaphragm dysfunction on admission to the intensive care unit. Prevalence, risk factors, and prognostic impact-a prospective study. Am J Respir Crit Care Med. (2013) 188:213–9. 10.1164/rccm.201209-1668OC 23641946

[33] Supinski GS Callahan LA. Diaphragm weakness in mechanically ventilated critically ill patients. Crit Care. (2013) 17:R120. 10.1186/cc12792 23786764 PMC3840677

[34] Sax H Contesse J Dubach P Reinhart WH. Creatine kinase MB during myocardial infarction: relationship to preexisting coronary heart disease and medication. Acta Cardiol. (1997) 52:423–30. 9428940

[35] Barthel BL Cox D Barbieri M Ziemba M Straub V Hoffman EP. Elevation of fast but not slow troponin I in the circulation of patients with Becker and Duchenne muscular dystrophy. Muscle Nerve. (2021) 64:43–9. 10.1002/mus.27222 33683712 PMC8362156

[36] Simpson JA Labugger R Collier C Brison RJ Iscoe S Van Eyk JE. Fast and slow skeletal troponin I in serum from patients with various skeletal muscle disorders: a pilot study. Clin Chem. (2005) 51:966–72. 10.1373/clinchem.2004.042671 15833785

[37] Dres M Dubé B-P Goligher E Vorona S Demiri S Morawiec E. Usefulness of parasternal intercostal muscle ultrasound during weaning from mechanical ventilation. Anesthesiology. (2020) 132:1114–25. 10.1097/ALN.0000000000003191 32084029

[38] Guttmann J Eberhard L Fabry B Bertschmann W Zeravik J Adolph M. Time constant/volume relationship of passive expiration in mechanically ventilated ARDS patients. Eur Respir J. (1995) 8:114–20. 10.1183/09031936.95.08010114 7744177

[39] Schepens T Goligher EC. Lung- and diaphragm-protective ventilation in acute respiratory distress syndrome: rationale and challenges. Anesthesiology. (2019) 130:620–33. 10.1097/ALN.0000000000002605 30844950

[40] Bertoni M Spadaro S Goligher EC. Monitoring patient respiratory effort during mechanical ventilation: lung and diaphragm-protective ventilation. Crit Care. (2020) 24:106. 10.1186/s13054-020-2777-y 32204729 PMC7092676

[41] Spadaro S Scaramuzzo G Volta CA. Can abdominal muscle ultrasonography during spontaneous breathing and cough predict reintubation in mechanically ventilated patients? Chest. (2021) 160:1163–4. 10.1016/j.chest.2021.07.006 34625161

