# Supplementary material for: Circulating Skeletal Troponin During Weaning From Mechanical Ventilation and Their Association to Diaphragmatic Function: A Pilot Study
Source: Front Med (Lausanne). 2021 Dec 22;8:770408. doi: 10.3389/fmed.2021.770408 (PMC8727747; doi:10.3389/fmed.2021.770408)
Supplement: Supplementary file 1 [file Data_Sheet_1.docx]

**Circulating skeletal troponin during weaning from mechanical ventilation and their association to diaphragmatic function: a pilot study**

Savino Spadaro, *^†^ ^1^ Francesca Dalla Corte, ^1,2 †^ Gaetano Scaramuzzo, ^1^ Salvatore Grasso,^3^ Gilda Cinnella,^4^Valentina Rosta,^5^ Valentina Chiavieri,^1^ Valentina Alvisi,^1^ Rosa Di Mussi,^3^ Carlo Alberto Volta,^1^ Tiziana Bellini,^5^ Alessandro Trentini.^5^

^†^ These authors have contributed equally to this work and share first authorship

ONLINE SUPPLEMENT

**Supplemental table 1. Correlation matrix between muscle biomarkers and respiratory effort parameters.**

| **Within subjects** | | | | |
| --- | --- | --- | --- | --- |
|  | **DE** | **TFdi** | **TEE** | **P_0.1_** |
| **ssTnI** | 0.056 (0.555) | 0.078 (0.407) | -0.083 (0.38) | 0.130 (0.265) |
| **fsTnI** | -0.035 (0.704) | 0.133 (0.15) | -0.014 (0.875) | -0.024 (0.83) |
| **CK** | 0.104 (0.264) | 0.130 (0.162) | -0.048 (0.611) | 0.031 (0.791) |
| **Myoglobin** | **0.464 (0.001)** | **0.462 (0.001)** | 0.073 (0.566) | 0.210 (0.163) |
| ***Between subjects*** | | | | |
|  | **DE** | **TFdi** | **TEE** | **P_0.1_** |
| **ssTnI** | **0.332 (0.038)** | -0.133 (0.499) | **0.445 (0.005)** | 0.236 (0.242) |
| **fsTnI** | **0.346 (0.027)** | 0.092 (0.623) | **0.400 (0.009)** | **0.473 (0.015)** |
| **CK** | 0.199 (0.196) | 0.104 (0.578) | **0.381 (0.016)** | **0.657 (0.002)** |
| **Myoglobin** | -0.161 (0.434) | -0.076 (0.753) | 0.164 (0.422) | 0.314 (0.196) |

Values within brackets represent the p-value. In bold are highlighted the significant correlations. DE: Diaphragmatic Excursion; TFdi: thickening fraction; TEE: Diaphragmatic thickness at end expiration; P_0.1_: airway occlusion pressure.

Supplemental table 2: Thickening fraction at day 3 and ICU outcome measures

|  | TFdi < 30% (33) | TFdi > 30 % (29) | P value |
| --- | --- | --- | --- |
| ICU mortality, n (%) | 6 (18.2%) | 6 (20.7%) | 0.8 |
| 28-days mortality, n (%) | 8 (24.2%) | 2 (6.9%) | 0.64 |
| ICU length of stay, days* | 14±13 | 19±13 | 0.34 |
| Tracheostomy rate, n (%) | 6 (18.2%) | 5 (17.2%) | 0.93 |
| Reintubation rate, n (%) | 4 (16%) | 2 (8.7%) | 0.67 |

**TF= thickening fraction. ICU= intensive care unit. Pearson Chi-square test; for *, independent T-Test.**

Supplemental table 3: Diaphragmatic displacement at day 3 and ICU outcome measures

|  | DE < 10 mm (32) | DE > 10 mm (30) | P value |
| --- | --- | --- | --- |
| ICU mortality, n (%) | 5 (15.6%) | 7 (23.3%) | 0.44 |
| 28-days mortality, n (%) | 5 (15.6%) | 5 (16.7%) | 0.64 |
| ICU length of stay, days* | 17±14 | 16±13 | 0.57 |
| Tracheostomy rate, n (%) | 6 (18.8%) | 5 (16.7%) | 1 |
| Reintubation rate, n (%) | 2 (7.7%) | 4 (18.2%) | 0.27 |

**DE= Diaphragmatic displacement. ICU= intensive care unit. Pearson Chi-square test; for *, independent T-Test. Pearson Chi-square test; for *, independent T-Test.**

**Supplemental table 4**: trend of diaphragmatic disfunction and thickening fraction during the three days of study.

|  | Day 1 | Day 2 | Day 3 | Mixed-effect analysis |
| --- | --- | --- | --- | --- |
| Diaphragmatic displacement (cm) | 1.2 [0.88-1.8] | 1.3 [0.78-1-7] | 1.0 [0.7-1.5]* | <0.0001 |
| Thickening fraction (%) | 0.38 [0.24-0.38] | 0.32 [0.24-0.4]* | 0.30 [0.22-0.4]* | <0.0001 |

Median [IQR]. Mixed effect analysis; * = different from day 1, Dunnett correction for multiple comparisons.

**Supplemental figure 1**: Kinetics of ssTnI and fsTnI in patient with normal diaphragm and diaphragmatic disfunction.


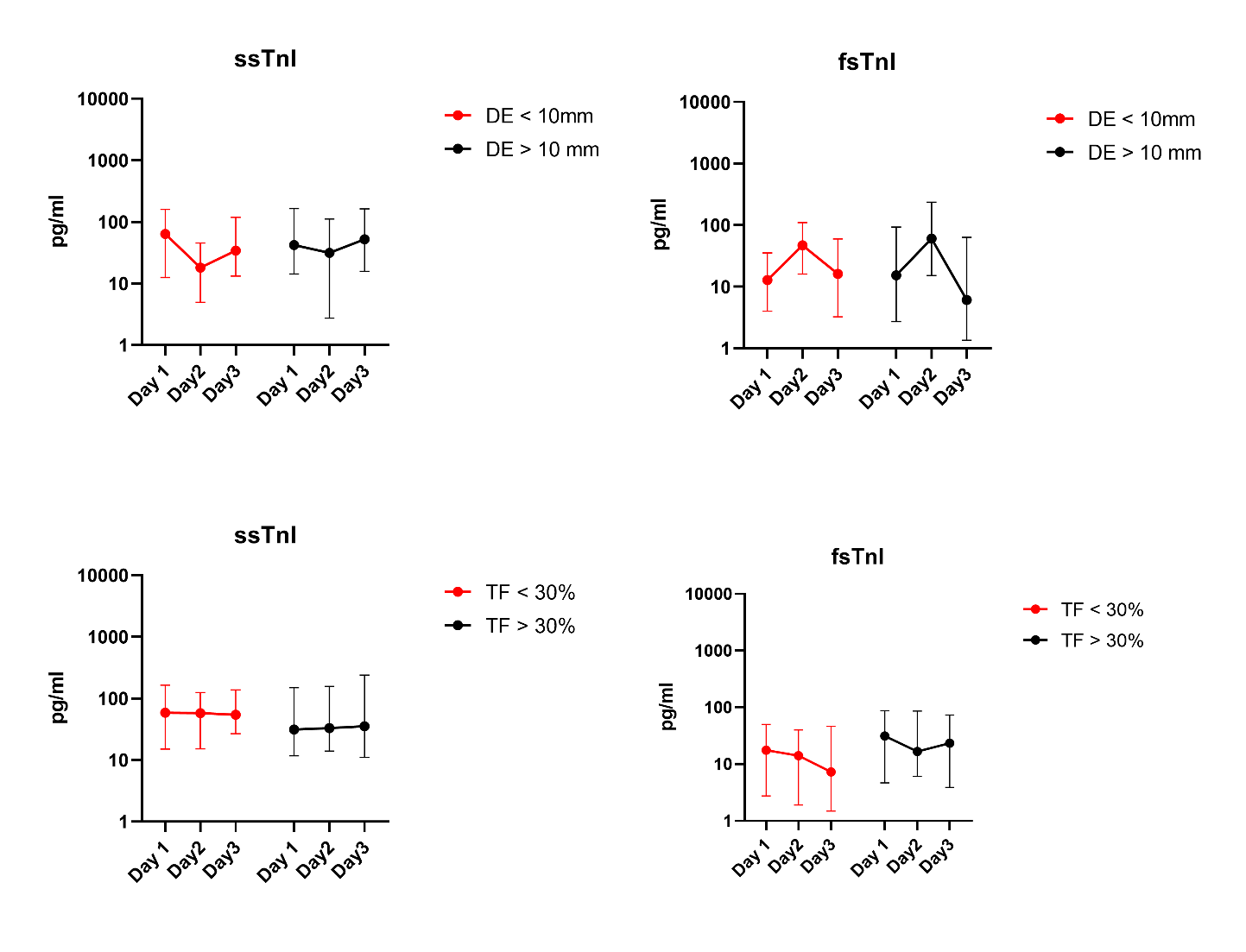


ssTnI= slow skeletal troponin isoform; fsTnI= fast skeletal troponin isoform; DE= diaphragmatic excursion; TF= thickening fraction. See the main manuscript for further details.
